# Characterization of the “gut microbiota-immunity axis” and microbial lipid metabolites in atrophic and potential celiac disease

**DOI:** 10.3389/fmicb.2022.886008

**Published:** 2022-09-30

**Authors:** Federica Ricci, Edda Russo, Daniela Renzi, Simone Baldi, Giulia Nannini, Gabriele Lami, Marta Menicatti, Marco Pallecchi, Gianluca Bartolucci, Elena Niccolai, Matteo Cerboneschi, Serena Smeazzetto, Matteo Ramazzotti, Amedeo Amedei, Antonino Salvatore Calabrò

**Affiliations:** ^1^Department of Biomedical, Experimental and Clinical Sciences “Mario Serio” University of Florence, Tuscany Regional Referral Center for Adult Celiac Disease, Florence, Italy; ^2^Department of Experimental and Clinical Medicine, University of Florence, Florence, Italy; ^3^Department of Neurosciences, Psychology, Drug Research and Child Health (NEUROFARBA), University of Florence, Florence, Italy; ^4^Department of Biomedical, Experimental and Clinical Sciences “Mario Serio” University of Florence, Florence, Italy

**Keywords:** potential celiac disease, celiac disease, microbiota, immune response, fatty acids, T cells, cytokines

## Abstract

**Introduction:**

Potential celiac disease (pCD) is characterized by genetic predisposition, positive anti-endomysial and anti-tissue transglutaminase antibodies, but a normal or almost normal jejunal mucosa (e.g., minor histological abnormalities without villous atrophy). To gain further insights into basic mechanisms involved in the development of intestinal villous atrophy, we evaluated and compared the microbial, lipid, and immunological signatures of pCD and atrophic CD (aCD).

**Materials and methods:**

This study included 17 aCD patients, 10 pCD patients, and 12 healthy controls (HC). Serum samples from all participants were collected to analyze free fatty acids (FFAs). Duodenal mucosa samples of aCD and pCD patients were taken to evaluate histology, tissue microbiota composition, and mucosal immune response.

**Results:**

We found no significant differences in the mucosa-associated microbiota composition of pCD and aCD patients. On the other hand, in pCD patients, the overall abundance of serum FFAs showed relevant and significant differences in comparison with aCD patients and HC. In detail, compared to HC, pCD patients displayed higher levels of propionic, butyric, valeric, 2-ethylhexanoic, tetradecanoic, hexadecanoic, and octadecanoic acids. Instead, aCD patients showed increased levels of propionic, isohexanoic, and 2-ethylhexanoic acids, and a lower abundance of isovaleric and 2-methylbutyricacids when compared to HC. In addition, compared to aCD patients, pCD patients showed a higher abundance of isobutyric and octadecanoic acid. Finally, the immunological analysis of duodenal biopsy revealed a lower percentage of CD4^+^ T lymphocytes in pCD infiltrate compared to that observed in aCD patients. The functional characterization of T cells documented a pro-inflammatory immune response in both aCD and pCD patients, but the pCD patients showed a higher percentage of Th0/Th17 and a lower percentage of Th1/Th17.

**Conclusion:**

The results of the present study show, for the first time, that the duodenal microbiota of patients with pCD does not differ substantially from that of aCD; however, serum FFAs and local T cells displayed a distinctive profile between pCD, aCD, and HC. In conclusion, our result may help to shed new light on the “gut microbiota-immunity axis,” lipid metabolites, and duodenal immune response in overt CD and pCD patients, opening new paradigms in understanding the pathogenesis behind CD progression.

## Introduction

Celiac disease (CD) is an immune-mediated systemic disorder elicited, in genetically susceptible individuals, by the ingestion of gluten and related prolamines present in barley and rye. CD is a common inflammatory disease that may affect not only the small intestine but also many extra-intestinal sites, with an estimated prevalence of about 1% in the European and North American populations (Karell et al., 2003; Ciccocioppo et al., 2005). Gluten acts in concert with the HLA-DQ2 or -DQ8 genes of predisposition, the mechanistically involved autoantigen tissue transglutaminase (TG2), and the activation of adaptive immune response (CD4^+^ T and B cells) leading to enterocyte damage and the development of villous atrophy, the histological hallmark of CD. In particular, in CD patients, incompletely digested gliadin peptides translocate, largely via trans-epithelial transport, from the intestinal lumen to the *lamina propria*, where they stimulate the release of TG2. Once secreted, the enzyme catalyzes the deamidation of specific glutamine in the gliadin peptides into glutamic acid, thus creating epitopes with higher affinity for the molecules DQ2/DQ8 of major histocompatibility complex class II (MHCII). Deamidated gluten peptides bind to HLA-DQ2 or -DQ8 expressed on antigen-presenting cells (APCs) and induce the activation of CD4^+^ T cells, which are responsible for intraepithelial lymphocyte (IEL) infiltration, crypt hyperplasia, and villous atrophy, and the subsequent production of anti-endomysial (EMA), anti-tissue transglutaminase (tTG2), and anti-gliadin (AGA) antibodies (Jabri and Sollid, 2009; Monteleone et al., 2010; Husby et al., 2012; Rubio-Tapia et al., 2012; van Bergen et al., 2015). Gluten-specific CD4 + T cells have also been involved as the primary driver of acute cytokine release and the onset of digestive symptoms after gluten ingestion. In addition, increasing evidence indicates that the induction of a gluten-specific adaptive CD4^+^ T-cell response must be preceded by the activation of the innate immune system and that mast cells, key players of the innate immune response, contribute to the pathogenesis of CD [as reviewed in Anderson (2020) and Frossi et al. (2019)].

The innate and adaptive immune responses, working in synergism, lead to villous atrophy and a variable combination of clinical manifestations, from the classical syndrome of malabsorption to light or even the absent of symptoms (Ludvigsson et al., 2013).

On the other hand, potential CD (pCD) is a condition characterized by the presence of positive CD serology and genetic susceptibility, but a normal (Marsh 0) or almost normal (Marsh 1–2) jejunal mucosa without villous atrophy (Troncone et al., 1996). The expression pCD was first proposed by Ferguson (Ferguson et al., 1993) for patients who do not have and never have had a jejunal biopsy consistent with overt CD and yet have immunological abnormalities similar to those found in CD. In detail, features that are good candidate markers of pCD include the presence of serum endomysial antibodies, a high count of villous intraepithelial lymphocytes, an increased density of IEL expressing γδ T-cell receptor, and signs of activated mucosal cell-mediated immunity, such as expression of CD25 and B7 by *lamina propria* mononuclear cells. Moreover, pCD is also characterized by enhanced expression of MHC class II molecules on the epithelium and on adhesion molecules in the lamina propria, all of which are enhanced by *in vitro* gluten challenge (Maiuri et al., 1996), a coeliac-like intestinal antibody pattern, and a positive rectal gluten challenge (Troncone et al., 1996).

This particular condition is usually considered a clinical challenge because, though pCD represents the early CD stage and its diagnostic criteria are clear, many questions are still unsettled and the results of the studies conducted so far are still conflicting (Kurppa et al., 2009; Tosco et al., 2011; Lionetti et al., 2012; Biagi et al., 2013; Zanini et al., 2013; Auricchio et al., 2014).

In addition to the involvement of the immune system, recent evidence reported several modifications in the intestinal tract microenvironment of CD patients, suggesting a role for gut microbiota in CD onset and persistence (Abdukhakimova et al., 2021). It is known that the gut bacteria play many fundamental roles for the host, such as the synthesis of many nutrients and metabolites (Gibiino et al., 2017; Thursby and Juge, 2017; Valdes et al., 2018), maintenance of the intestinal epithelial integrity (Khosravi and Mazmanian, 2013), and notably, the modulation of immune responses (Brestoff and Artis, 2013; Woo and Alenghat, 2017).

Furthermore, the production of short-chain fatty acids (SCFAs), the major bacterial fermentative end products, reflects the intestinal microbiota composition and activity. In particular, given that SCFAs are crucial to maintaining the host’s normal gut physiology and metabolic functions and that a part of them enters the systemic circulation (den Besten et al., 2013), it is tempting to speculate that the gut microbiota could be also related to free fatty acid (FFA) levels. FFAs, classified as short-chain fatty acids (SCFAs), medium-chain fatty acids (MCFAs), and long-chain fatty acids (LCFAs), derive from microbial and host metabolism and, especially MCFAs and LCFAs, are introduced into the diet (i.e., milk and dairy products). Since FFAs modulate the production of chemokines and cytokines (Frommer et al., 2015; Honda et al., 2015; Hung et al., 2015), an altered FFA profile has been associated with the risk of developing a range of disorders in which the immune system is involved, including CD (Aghdassi et al., 2011; Nishi et al., 2014; van Hees et al., 2014; Dai et al., 2015; Rodriguez-Carrio et al., 2016).

Therefore, given that pCD is a valuable biological model of the pathway leading to small intestinal mucosal damage in genetically predisposed subjects, our present study aimed to evaluate whether microbial, lipid, and immunological signatures could better characterize pCD from the overt CD condition. In addition, we also evaluated whether and how immunological peculiarity affects the composition of the duodenal microbiota and its metabolic activity, opening new paradigms in understanding the basic mechanisms involved in the development of small intestinal villous atrophy.

## Materials and methods

### Patient recruitment

Twenty-seven CD patients with positive IgA anti-endomysial (EMA) and anti-tissue transglutaminase (tTGA) antibodies consecutively observed at the Tuscany Regional Referral Center for adult CD were enrolled in this observational study from January 2018 to December 2019. Twelve healthy controls, with negative EMA and tTGA antibodies, and without any other clinical problems, were enrolled among the internal staff participating in the study.

Inclusion criteria were as follows: Age between 18 and 70 years, absence of any form of immunodeficiency, in particular selective IgA deficiency, and patients resident in Tuscany who, in the past 5 years, have not made any trips to countries outside Europe. This last criterion is very relevant for the study of the composition of intestinal microbiota. All patients recruited received adequate information on the study, the objectives, and how they will have to provide the organic samples for analysis. Each patient signed and dated the informed consent.

Exclusion criteria were as follows: Treatment with antibiotics or probiotics during the previous 2 months, acute gastrointestinal infections 1 month before the enrolment, pregnant and breastfeeding women (ongoing or scheduled for the next 48 weeks), the concomitant presence of established malignant neoplasms or chronic inflammatory bowel diseases (Crohn’s disease and ulcerative colitis), and patients who have used immunosuppressive drugs in the previous 3 months.

#### Patient classification

According to mucosal histology, patients were divided into two groups: aCD (5 men, 12 women, mean age 35.6 years, age range 17–56 years) and pCD (two men, 8 women, mean age 38.8 years, age range 21–50 years). In agreement with the definition of Troncone (Troncone et al., 1996), we decided to include cases with Marsh 2 in the pCD group, given the absence of villous atrophy in these patients. Moreover, 12 healthy controls (five men, seven women, mean age 33.6 years, age range 23–50 years), with negative EMA and tTGA antibodies, served as a control group (HC). For ethical reasons, esophagogastroduodenoscopy with small bowel biopsies was not performed in this group. All the study participants were on a gluten-containing diet and did not consume any drug at the time of examination.

#### Sample collection

After an overnight fast, venous blood samples were collected, and three serum aliquots per patient were immediately frozen and stored at -80°C until further use. All patients underwent an esophagogastroduodenoscopy, and 4–6 small bowel biopsies were taken from the distal part of the duodenum. Small intestinal mucosal damage was graduated according to the classification of Marsh modified by Oberhuber et al. (1999).

### Antibody testing

The tTGA levels were measured by a commercially available enzyme-linked immunosorbent assay kit (EutTG, Eurospital, Trieste, Italy) that employs human recombinant tTG as antigen. EMA antibodies were determined by indirect immunofluorescence, using tissue sections of monkey esophagus (Eurospital) as previously reported (Vignoli et al., 2019).

### Generation of t-cell clones from intestinal infiltrating lymphocytes

Duodenal mucosa samples were collected in RPMI 1640 culture medium (EuroClone, Italy) and dissociated with the Tumor Dissociation Kit, human (Miltenyi Biotech, UK) in combination with the gentleMACS™ Octo Dissociator (Miltenyi Biotech, Germany) to isolate tissue-infiltrating lymphocytes (TILs). TILs were magnetically isolated with anti-human CD3^+^ microbeads (MiltenyiBiotec, UK) using the AutoMACS Pro Separator (Miltenyi Biotech, Germany) and cloned under limiting dilution. Single T-cell clones (Tcc) were seeded in microwells (0.3 cells/well) containing RPMI 1640 supplemented with 10% FBS HyClone (Hyclone Laboratories, South Logan, Uthan), in the presence of 2 × 10^6^ irradiated (9,000 rad) peripheral blood mononuclear cells (PBMCs), phytohemagglutinin (PHA, 0.5% vol/vol; EuroClone, Italy), and recombinant human interleukin-2 (IL-2, 50 U/ml; Eurocetus, Italy). At weekly intervals, 2 × 10^6^ irradiated PBMCs and IL-2 were added to each micro-culture to maintain the expansion of growing clones.

### Phenotypic and functional characterization of isolated T-cell clones

The expression of Tcc surface markers (CD4 and CD8) was analyzed by flow cytometry using a BD FACSCanto*™* II, and a total of 10^4^ events for each sample was acquired. To assess cytokine profile, Tcc markers were resuspended at a concentration of 10^6^ cells/ml medium and cultured for 48 h in the presence of PHA (1%). Cell-free supernatants were collected and assayed in duplicate for IFN-γ, IL-4, IL-17, and IL-10 content by commercial ELISA kits (BioLegend, San Diego). The supernatants presenting cytokine levels that were 5 SD above the mean levels of control supernatants derived from irradiated antigen-presenting cells alone were regarded as positive. Based on the cytokine profile and the CD4/CD8 expression, we divided the Tcc into the following groups: Th1-Tc1 (only IFN-γ), Th2-Tc2 (only IL-4), Th17-Tc17 (only IL-17), Treg-Tcreg (only IL-10), Th0-Tc0 (IL-4 + IFN-γ), Th1/Th17-Tc1/Tc17 (IFN-γ + IL-17), and Th0/Th17-Tc0/Tc17 (IL-4 + IFN-γ + IL-17).

### Deoxyribonucleic acid extraction and bioinformatics analysis of 16S ribosomal ribonucleic acid

DNA extraction and bioinformatics analysis of 16S rRNA were performed as previously described (Niccolai et al., 2020), Briefly, total DNA was extracted from duodenal mucosa samples of aCD and pCD patients using the DNeasyPowerLyzerPowerSoil Kit (Qiagen, Hilden, Germany) from frozen samples (-80°C) according to the manufacturer’s instructions. The quality and quantity of extracted DNA were assessed using the Qubit Fluorometer (Thermo Fisher Scientific) and then genomic DNA was frozen at -20°C.

Extracted DNA samples were sent to NEXT Genomics (Sesto Fiorentino, Italy) where amplicons of the variable V3–V4 region of the bacterial 16s rRNA gene were sequenced in paired-end mode (2 × 300 cycles) on the Illumina MiSeq platform, according to the Illumina 16S Metagenomic Sequencing Library Preparation protocol (Pagliai et al., 2020; Russo et al., 2021). Raw sequences were processed following the software pipeline MICCA (Albanese et al., 2015). Paired-end reads were assembled using the “mergepairs” command, maintaining a minimum overlap of 100 bp and an edit distance in the maximum overlap of 32 bp. Subsequently, the sequences were cut with the “trim” command in order to remove the primers and eventually eliminate the reads with imperfections in primer sequences. All the reads with a length lower than 350 bp and with an error rate higher than or equal to 0.5 were removed with the “filter” command. Cleaned reads were eventually merged into a single file with the “merge” command and transformed into a fasta file. The OTUs were generated using the “otu” command in “denovo_greedy” mode, setting a 97% identity and performing an automatic removal of chimeras with the “-c” option. The longest sequence of each OTU was used for the taxonomic assignment using the “classify” command in “rdp” mode, i.e., using the RDP Bayesian classifier (10.1093/nar/gki038) that is able to obtain classification and confidence for taxonomic ranks up to genus level.

### Microbial community analysis

Statistical analyses on the bacterial community were performed in R (R Development Core Team, 2014) with the help of the packages phyloseq 1.26.1 (McMurdie and Holmes, 2013), DESeq2 1.22.2 (Love et al., 2014), and other packages satisfying their dependencies, in particular vegan 2.5–5 (Willis and Bunge, 2015). Rarefaction analysis on OTUs was performed using the function rarecurve (step 50 reads), further processed to highlight saturated samples (arbitrarily defined as saturated samples with a final slope in the rarefaction curve with an increment in OTU number per reads < 1e-2). For the cluster analysis (complete clustering on Euclidean distance) of the entire community, the OTU table was first normalized using the total OTU counts of each sample and then adjusted using square root transformation. The coverage was calculated by Good’s estimator using the formula: (1 - n/N) × 100, where n is the number of sequences found once in a sample (singletons), and N is the total number of sequences in that sample.

Richness, Shannon, Chao 1, and evenness indices were used to characterize the ecological properties of each sample using the function estimate_richness from phyloseq (McMurdie and Holmes, 2013). The evenness index was calculated using the formula E = S/log(R), where S is the Shannon diversity index and R is the number of OTUs in the sample. Differences in all indices between overt CD and pCD were tested using a paired Wilcoxon signed-rank test. The differential analysis of abundance at the OTUs, as well as at the different taxonomic ranks (created using the tax_glom function in phyloseq), was performed with DESeq2 (Love et al., 2014).

### Evaluation of free fatty acids by gas chromatography-mass spectrometry analysis

The analysis of FFAs was performed by Agilent gas chromatography-mass spectrometry (GC-MS) system composed of 5971 single quadrupole mass spectrometer, 5890 gas-chromatograph, and 7673 autosampler, with a dedicated previously described protocol (Baldi et al., 2021). The chemicals, GC-MS conditions, and calibration parameters were reported in supporting information SX. Just before the analysis, each sample was thawed. The FFAs were extracted as follows: an aliquot of 300 μl of serum sample was added to 10 μl of ISTD mixture, 100 μl of tert-butyl methyl ether, and 20 μl of 6 M HCl + 0.5 M NaCl solution in 0.5 ml centrifuge tube. Afterward, each tube was stirred in a vortex for 2 min, centrifuged at 10,000 rpm for 5 min, and finally, the solvent layer was transferred to a vial with a microvolume insert and analyzed.

### Statistical analysis

The statistical analysis of immunological and FFAs data was performed using GraphPad Prism (v. 5) software, and the data were expressed as the mean ± standard deviation (SD). The comparisons between aCD, pCD patients, and HC were evaluated with the Kruskal–Wallis test, while the comparisons between the Tcc groups were evaluated with the two-tailed Chi-square test or Fisher’s exact test, when appropriate (in detail, when ≤ 20% of cell counts were less than 5, we used the Chi-square test; if > 20% of expected cell counts were less than 5, we used Fisher’s exact test). *P*-values < 0.05 were considered statistically significant.

## Results

### Characteristics of the study population

Patients were divided into two groups: aCD (5 men, 12 women, mean age 35.6 years, age range 17–56 years) and pCD (2 men, 8 women, mean age 38.8 years, age range 21–50 years), in accordance with mucosal histology. In more detail, nine aCD patients had partial villous atrophy (Marsh 3 A), six subtotal villous atrophy (Marsh 3B), and two total villous atrophy (Marsh 3C); among pCD patients, histological examination revealed an apparently normal mucosa (Marsh 0) in three subjects, just an increase in intraepithelial lymphocytes (Marsh 1) in five, and increased intraepithelial lymphocytes coupled with crypt hyperplasia (Marsh 2) in two patients (Table 1). Twelve healthy controls (5 men, 7 women, mean age 33.6 years, age range 23–50 years), with negative EMA and tTGA antibodies, served as a control group (HC).

**TABLE 1:** Patients’ clinical parameters.

Patients	Codes	Age	Sex	Anti-tTG IgA (U/ml)	Anti-EMA-IgA	Diagnosis	Histology (grade marsh)
Patient 1	IMM2	18	M	24.1	Positive	aCD	3C
Patient 2	IMM5	21	M	22.2	Positive	aCD	3B
Patient 3	IMM6	24	F	97.8	Positive	aCD	3A
Patient 4	IMM8	28	F	28.0	Positive	aCD	3A
Patient 5	IMM9	54	F	14.4	Weakly positive	pCD	0
Patient 6	IMM10	21	F	13.8	Positive	pCD	1
Patient 7	IMM11	19	F	23.4	Positive	aCD	3A
Patient 8	IMM13	29	F	30.9	Positive	pCD	2
Patient 9	IMM22	31	F	>100	Positive	pCD	0
Patient 10	IMM26	28	F	>100	Positive	aCD	3A
Patient 11	IMM27	52	F	>100	Positive	aCD + HD	3A
Patient 12	IMM31	45	F	>100	Positive	aCD	3B
Patient 13	IMM35	41	F	95.3	Positive	aCD	3A
Patient 14	IMM39	48	M	>100	Positive	pCD + DH	0
Patient 15	IMM40	54	F	>100	Positive	aCD	3B
Patient 16	IMM41	19	F	12.2	Positive	aCD	3A
Patient 17	IMM48	17	M	>100	Positive	aCD	3C
Patient 18	IMM51	40	M	17.0	Positive	aCD	3B
Patient 19	IMM59	41	F	19.7	Positive	pCD + HD	2
Patient 20	IMM60	43	F	30.4	Positive	pCD	1
Patient 21	IMM61	30	F	11.2	Weakly positive	pCD	1
Patient 22	IMM68	41	F	47.0	Positive	aCD	3A
Patient 23	IMM70	41	F	19.1	Weakly positive	pCD	1
Patient 24	IMM73	46	F	92.0	Positive	aCD	3B
Patient 25	IMM82	56	M	32.0	Positive	aCD + HD	3A
Patient 26	IMM108	50	M	13.5	Weakly positive	pCD	1
Patient 27	IMM109	56	M	>100	Positive	aCD	3B

DH, herpetiform dermatitis.

### Analysis of microbiota composition and free fatty acids of atrophic CD and potential celiac disease groups

#### Comparison of tissue microbiota composition between atrophic CD and potential celiac disease patients

**FIGURE 1:**
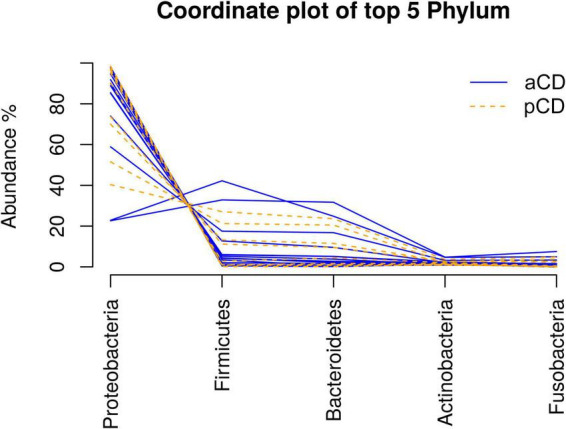
Taxonomic composition of aCD and pCD patients’ microbiota Coordinate plot showing the relative abundance of the five most abundant phyla in each aCD and pCD duodenal biopsy.

**FIGURE 2:**
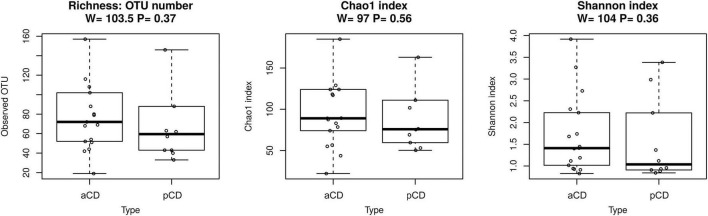
Boxplots reporting alpha diversity indices (respectively, Richness, Shannon index, and Chao1 index) in aCD and pCD samples. Alpha diversity indexes are composite indexes reflecting abundance and consistency. Boxes represent the interquartile range (IQR) between the first and third quartiles (25th and 75th percentiles, respectively), and the horizontal line inside the box defines the median. Whiskers represent the lowest and highest values within 1.5 times the IQR from the first and third quartiles, respectively. *P*-values less than 0.05 were considered statistically significant.

**FIGURE 3:**
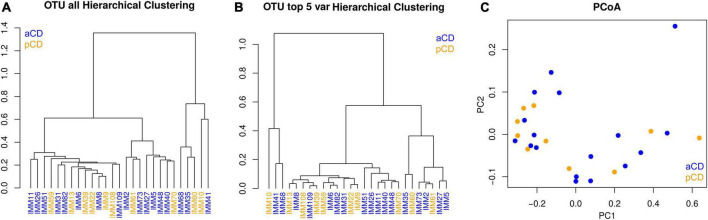
Multivariate representations of the entire sample set. **(A)** Complete hierarchical clustering based on Euclidean distance of all identified OTUs, **(B)** complete hierarchical clustering based on Euclidean distance of the top five most informative OTUs, **(C)** principal coordinate analysis (PCoA) using Bray-Curtis dissimilarity as a distance metric.

We first analyzed the microbiota composition of duodenal mucosa samples obtained for both CD and pCD patients. We obtained a total of 737,729 reads, and after all preprocessing steps (pair merging, trimming, quality filtering, and chimeras removal), a total of 523,825 reads were available for further analysis (Table 2, see Supplementary material for details). As reported in Figure 1, the analysis of the taxonomic composition revealed that more than 99% of the sequences collected could be ascribed to the five most representative phyla: *Proteobacteria* (72.98%), *Firmicutes* (11.92%), *Bacteroidetes* (10.36%), *Actinobacteria* (2.33%), and *Fusobacteria* (2.16%). As shown in Figure 2, no significant difference was reported for richness and Shannon and Chao1 alpha diversity indices. Moreover, in order to investigate the similarity of patients in terms of taxonomy abundance profiles, we evaluated PCoA and NMDS and completed the hierarchical clustering using several data transformations and the Bray-Curtis dissimilarity as a distance metric. Surprisingly and despite our efforts, no evident bacterial groupings were observable between aCD and pCD patients. As evidence of our fine analysis, in Figure 3, we reported several multivariate plots built upon square root transformed percent abundances, namely, a) hierarchical clustering of Euclidean distance, b) hierarchical clustering of the top five most abundant taxa only, and c) a PCoA of Bray-Curtis distance.

**TABLE 2:** Summary of the taxonomic analysis of the obtained OTUs.

Rank	Counts	Reads	% Reads	OTU	% OTU
Phylum	15	515,816	99.97771	320	99.37888
Class	24	515,570	99.93003	315	97.82609
Order	37	515,570	99.93003	315	97.82609
Family	69	515,264	99.87072	303	94.09938
Genus	116	508,646	98.58799	256	79.50311

#### Evaluation of serum free fatty acids profiles

**FIGURE 4:**
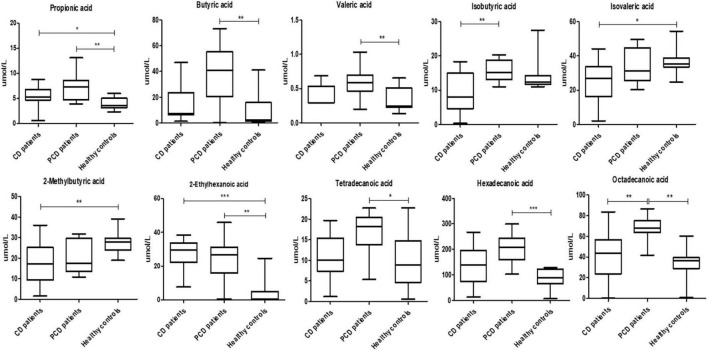
Boxplots representing the overall abundance of FFAs in aCD patients, pCD patients, and healthy controls (μmol/L). *P*-values of the intergroup comparisons were assessed with Kruskal–Wallis test. *P*-values less than 0.05 were considered statistically significant. The asterisks * represent *p*-values, *p < 0.05, **p < 0.01, ***p < 0.001.

We evaluated the metabolic profile of the patients performing the qualitative and quantitative analysis of serum FFAs, namely, linear SCFAs (acetic, propionic, butyric, and valeric acids), branched SCFAs (isobutyric, isovaleric, 2-ethylhexanoic, 2-methylbutyric, and cyclohexanoic acids), ethylhexanoic and cyclohexanoic are MCFAs and not branched SCFAs (hexanoic, heptanoic, octanoic, nonanoic, decanoic, and dodecanoic acids), and LCFAs (tetradecanoic, hexadecanoic, and octadecanoic acids), between aCD patients, pCD patients, and HC (Figure 4).

In detail, comparing aCD and pCD patients, the latter showed significantly higher levels of isobutyric (*p* < 0.01) and octadecanoic acids (*p* < 0.01). Moreover, pCD vs aCD patients displayed a higher abundance of propionic (*p* < 0.01), butyric (*p* < 0.01), valeric (*p* < 0.01), 2-ethylhexanoic (*p* < 0.01), tetradecanoic (*p* < 0.05), hexadecanoic (*p* < 0.001), and octadecanoic (*p* < 0.01) acids compared to HC. Finally, aCD patients, in comparison with the healthy controls, showed increased levels of propionic (*p* < 0.05) and 2-ethylhexanoic (*p* < 0.001) acids and a lower abundance of isovaleric (*p* < 0.05) and 2-methylbutyric (*p* < 0.01) acids (Table 3).

**TABLE 3:** SCFA, MCFA, and LCFA abundance in aCD and pCD patients and HC.

SCFAs (μ mol/L)	aCD	pCD	HC
Acetic acid	119.21 ± 57.46	121.91 ± 26.32	127.17 ± 48.38
Propionic acid	5.07 ± 2.04	7.37 ± 2.82	4.10 ± 1.19
Butyric acid	5.93 ± 5.46	37.61 ± 23.04	11.50 ± 16.18
Isobutyric acid	6.06 ± 4.18	15.73 ± 3.30	12.36 ± 1.15
2-Methylbutyric acid	13.85 ± 6.58	20.26 ± 7.98	26.44 ± 4.16
Isovaleric acid	19.88 ± 9.32	33.28 ± 9.93	35.10 ± 4.99
Valeric acid	0.33 ± 0.12	0.58 ± 0.22	0.35 ± 0.18
**MCFAs (μmol/L)**			
Hexanoic acid	1.79 ± 0.92	2.16 ± 1.02	2.95 ± 1.27
Isohexanoic acid	0.09 ± 0.03	0.09 ± 0.03	0.06 ± 0.01
2-Ethylexanoic acid	29.77 ± 4.36	24.21 ± 13.87	5.31 ± 9.27
Cyclohexanoic acid	0.29 ± 0.13	0.43 ± 0.06	0.31 ± 0.01
Heptanoic acid	0.35 ± 0.29	0.08 ± 0.03	0.09 ± 0.08
Octanoic acid	1.43 ± 0.33	1.43 ± 0.33	1.18 ± 1.57
Non-anoic acid	0.06 ± 0.03	0.06 ± 0.03	0.05 ± 0.01
Decanoic acid	2.42 ± 0.92	2.42 ± 0.92	2.45 ± 2.60
Dodecanoic acid	3.45 ± 1.75	3.45 ± 1.75	2.19 ± 2.48
**LCFAs (μmol/L)**			
Tetradecanoic acid	16.61 ± 5.30	16.61 ± 5.30	8.72 ± 7.27
Hexadecanoic acid	204.93 ± 61.25	204.93 ± 61.25	76.13 ± 41.66
Octadecanoic acid	68.40 ± 12.77	68.40 ± 12.77	30.84 ± 16.85

Data are presented as median (standard deviation). aCD, atropic celiac disease; pCD, potential celiac disease; HC, healthy control; SCFAs, short-chain fatty acids; MCFAs, medium-chain fatty acids; LCFAs, long-chain fatty acids.

### Characterization of mucosal infiltrating T cells in atrophic CD and potential celiac disease patients

We evaluated differences in the T-cell infiltration using biopsies of the duodenal mucosa of aCD and pCD patients; for ethical reasons, we were not able to obtain duodenal biopsies from HC to be used to compare the results.

We cloned and expanded *in vivo*-activated TILs, obtaining a total number of 267 T-cell clones: 206 Tcc from aCD patients and 61 Tcc from pCD patients.

**FIGURE 5:**
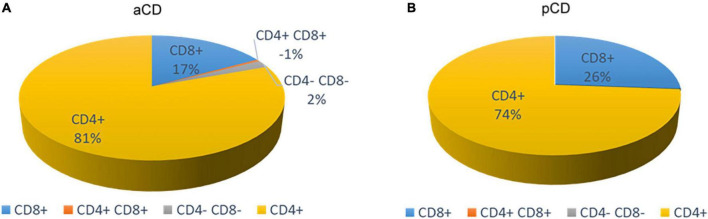
Percentage of CD4 + and CD8 + T-cell clones, respectively, obtained from the duodenal mucosa of patients with atrophic CD **(A)** and potential CD **(B)**.

The evaluation of the surface markers showed a prevalence of CD4^+^Tcc in both patient subgroups with a lower percentage in pCD patients [pCD vs aCD: 73.8% (45/61) vs 80.6% (166/206)]. Moreover, the analysis showed 26.2% (16/61) CD8^+^Tcc in the duodenal mucosa of pCD patients vs 17% (35/206) CD8^+^Tcc in the duodenal mucosa of aCD patients, 0.5% (1/206) CD4^+^/CD8^+^Tcc, and an interesting percentage (1.9%, 4/206) of CD4^–^/CD8^–^Tcc only in the gut mucosa of aCD patients (Figure 5).

**FIGURE 6:**
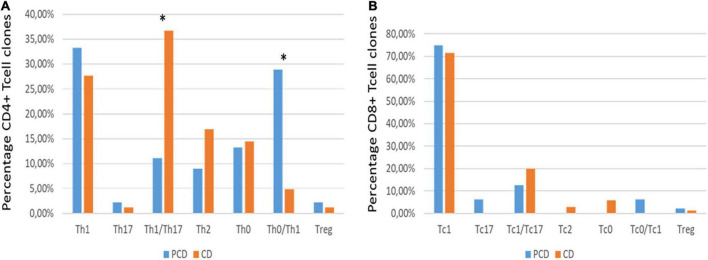
**(A)** The cytokine phenotype percentage of CD4^+^ T-cell clones obtained from the duodenal mucosa of patients with atrophic CD (orange) and potential CD (blue). *P*-values of the intergroup comparisons were assessed with Fisher’s exact test. *P*-values less than 0.05 were considered statistically significant. The asterisks * represent *p*-values, *p < 0.05. **(B)** The cytokine phenotype percentage distribution of CD8^+^ T-cell clones obtained from the duodenal mucosa of patients with atrophic CD (orange) and potential CD (blue).

Based on the cytokine profile of CD4^+^ Tcc, most of them produced IFN-γ [pCD vs aCD: 86.6% (39/45) vs 80.7% (134/166)] (Two-tailed Chi-square test, *p* = 0.3574). In addition, the fine analysis of the CD4^+^ T-cell subsets (Figure 6A) revealed that the duodenal mucosa of pCD patients showed lower percentages of Th1/Th17 [pCD vs aCD: 11.1% (5/45) vs 36.7% (61/166)] (*p* = 0.001), Th0 [pCD: 13.3% (6/45) vs 14.5% (24/166)] (*p* = 0.8481), Th2 [pCD vs aCD: 9% (4/45) vs 16.9% (28/166)] (*p* = 0.2437), and higher percentages of Th1 [pCD vs aCD: 33.3% (15/45) vs 24.7% (41/166)] (*p* = 0.2446), Th0/Th17 [pCD vs aCD: 28.9% (13/45) vs 4.8% (8/166)] (Two-tailed Chi-square test, *p* < 0.0001), Th17 [pCD vs aCD: 2.2% (1/45) vs 1.2% (2/166)] (*p* = 0.5149), and Treg [pCD vs aCD: 2.2% (1/45) vs 1.2% (2/166)] (*p* = 0.5149).

Regarding the cytokine profile of CD8 + population, we documented again that the majority produced IFN-γ [pCD vs aCD: 93.75% (15/16) vs 97.1% (34/35)] (*p* = 0.5333).

In addition, the analysis of CD8^+^ T-cell subsects (Figure 6B) showed in the duodenal mucosa of pCD patients lower percentages of Tc1/Tc17 [pCD vs aCD: 12.5% (2/16) vs 20% (7/35)] (*p* = 0.7012), Tc0 [pCD vs aCD: 0% vs 5.7% (2/35)] (*p* = 1.0000), Tc2 [pCD vs aCD: 0% vs 2.9% (1/35)] (*p* = 1.0000), and higher percentages of Tc1 [pCD vs aCD: 75% (12/16) vs 71.4% (25/35)] (*p* = 0.7909), Tc0/Tc17 [pCD vs aCD: 6.25% (1/16) vs 0%] (*p* = 0.3137), and Tc17 [pCD vs aCD: 6.25% (1/16) vs 0%] (*p* = 0.3137).

## Discussion

In this original study, we show for the first time that the duodenal microbiota of patients with pCD does not differ substantially from that of aCD. On the other hand, we observed differences in the local immune T-cell population (increased Th0/Th17 and reduced Th1/Th17 in pCD), while serum FFAs displayed a distinctive profile between pCD, aCD, and HC.

Although great advances have been made in understanding the role of CD adaptive immune mechanisms in response to gluten peptides, and their crosstalk with the intestinal microbiota, the complete set of pathogenic events causing the development of tissue lesions remains unclear. In our study, therefore, we explored for the first time the complex crosstalk between the microbiota, lipid metabolites, and the T-cell profile in both aCD and pCD patients, to better characterize aspects still unclear in the pCD condition and assess further significant differences between the two faces of celiac disease.

First, we compared the mucosal microbiota composition in the duodenal biopsies of aCD and pCD patients. Since most studies have been conducted using both different sequencing methods and inhomogeneous sampling, the findings on the gut microbiota composition of aCD patients are highly heterogeneous and sometimes contrasting.

Previous studies (Nistal et al., 2012; Cheng et al., 2013) showed that the most abundant bacterial phyla in CD adults were *Firmicutes, Bacteroidetes*, and *Actinobacteria*, whereas CD children displayed higher abundances of *Proteobacteria, Bacteroidetes*, and *Actinobacteria*. In accordance with these findings, we observed an abundance of *Firmicutes*, *Bacteroidetes*, and *Actinobacteria* in both CD and pCD patients; however, in contrast with the previous results, we observed high percentages of *Proteobacteria* (72.98%) and *Fusobacteria* in adult CD and pCD patients.

The high abundance of Gram-negative *Proteobacteria* may result in altered exposure of intestinal epithelial cells to bacterial lipopolysaccharides (LPS) and some metabolites, such as SCFAs (Akobeng et al., 2020). Intriguingly, no statistically different microbial taxa or bacterial groupings were displayed between the two patients’ cohorts, suggesting the existence of a similar microbial consortium in both conditions.

Even though we demonstrated the presence of a stable microbiota composition between overt CD and pCD patients, based on our previous metabolic results (Bertini et al., 2009; Bernini et al., 2011), we hypothesized that the functional metabolic activity of gut microbiota could be differently regulated between the two pathologic conditions. Indeed, in one of our previous studies, by employing a non-invasive metabonomic approach, we have first shown that CD has a well-defined metabonomic signature (Bertini et al., 2009). Notably, sera of CD patients were characterized by lower levels of several metabolites, such as amino acids, lipids, pyruvate, and choline, and by higher levels of glucose and 3-hydroxybutyric acid, while urine samples showed altered levels of, among others, indoxyl sulfate, meta-[hydroxyphenyl]propionic acid, and phenylacetylglycine. Moreover, in another study addressed to pCD and CD patients as well as HC, we observed, examining serum and urine samples, that pCD largely shares the metabonomic signature of aCD (Bernini et al., 2011). Most metabolites found to be significantly different between control and CD subjects were also altered in pCD (Bernini et al., 2011).

Regarding the free fatty acids, altered circulating levels of SCFAs, MCFAs, and LCFAs and LCFAs, have been related to CD (Niccolai et al., 2017; Baldi et al., 2021). In our previous study, we compared the serum FFA profile of patients with CD, adenomatous polyposis (AP), and colorectal cancer (CRC) to HC (Baldi et al., 2021). We observed that HC showed a different composition of FFAs as compared to CRC, AP, and CD patients. Moreover, we demonstrated a strong positive association between CD and butyric acid, suggesting for the first time that it could represent a potential biomarker for CD screening. However, no information addressing the serum SCFA composition of pCD patients is currently available. So, as they provide information on the general health status of both the host and its intestinal microbial ecosystem, we evaluated the abundance of each circulating FFA. In detail, compared to HC, pCD patients displayed higher levels of propionic, butyric, valeric, 2-ethylhexanoic, tetradecanoic, hexadecanoic, and octadecanoic acids. Propionic, butyric, and valeric acids are the most common microbiota-produced SCFAs that, as well established, exert positive metabolic effects on enterocytes, act as tumor suppressor agents, and regulate host gene expression *via* histone deacetylase inhibition (HDAC; Nankova et al., 2014; Yuille et al., 2018).

Moreover and notably, butyric acid is involved in Tregs differentiation (Kanauchi et al., 1999; Atarashi et al., 2013) and in the maintenance of the gastrointestinal epithelial barrier integrity (Braniste et al., 2014; Buscarinu et al., 2017; Mizuno et al., 2017), thus preventing microbial translocation and LPS-driven triggering of TLR4-mediated signaling.

In contrast to SCFAs, MCFAs and LCFAs arise mostly from dietary triglycerides and are important regulators of energy metabolism, gene expression, and immune processes (Schonfeld and Wojtczak, 2016; Pinkosky et al., 2020). In particular, they can antagonize the anti-inflammatory activities of SCFAs, as they support the Th1 and Th17 differentiation, so both MCFAs and LCFAs could be involved in the onset and persistence of duodenal inflammation in pCD patients, compared to HC.

In aCD patients, the results of our study showed increased levels of propionic, isohexanoic, and 2-ethylhexanoic acids, and a lower abundance of isovaleric and 2-methylbutyric acids as compared to HC. In addition, we found that, compared to aCD patients, pCD patients showed higher abundances of isobutyric and octadecanoic acid. Isobutyric acid is a branched SCFA produced by valine and leucine fermentation, and a high abundance of this acid indicates an increased amino acid fermentation. So, this finding suggests that, even if the composition of the duodenal microbiota is almost similar, aCD and pCD patients displayed a different microbial functional activity, which deserves to be deepened in future studies.

As there is a documented mutual interplay between the microbiome and the immune response, to assess whether and how immunological peculiarity affects the composition of the duodenal microbiota and its metabolic activity, we also evaluated the cellular immune profile in aCD and pCD patients. In detail, the phenotypic characterization of T cells revealed, in agreement with previous studies, a lower percentage of CD4^+^ T cells in pCD patients compared to CD patients. It has been shown that CD4^+^ T cells play a key role in the inflammatory response triggered by gluten, by driving and orchestrating the gut inflammation and tissue destruction in human celiac disease (Voisine and Abadie, 2021).

Moreover, in accordance with Vitale et al., pCD patients showed a high percentage of T cells co-producing IL-17, IL-4, and IFN-γ (Th0/Th17 *p* < 0.00005, Tc0/Tc17; Vitale et al., 2019). Notably, IL-17 is a pro-inflammatory cytokine, but a number of studies underlined its potential involvement in the prevention of gut mucosa inflammation (Mucida and Salek-Ardakani, 2009; Cukrowska et al., 2017). In addition, as compared to atrophic CD, pCD patients showed lower percentages of T cells co-producing IFN-γ and IL-17 (Th1/Th17 *p* < 0.001). Of note, the Th1/Th17 subpopulation promotes damage to the enterocytes leading to the atrophy of the intestinal villi (Cukrowska et al., 2017). We hypothesize that the increase of Th0/Th17 linked with the decrease of Th1/Th17 might contrast the inflammation, contributing to preventing mucosal damage in the pCD patients. However, whether and how this immunological peculiarity can affect the composition of the duodenal microbiota and its metabolic activity remains unknown.

Therefore, although our exploratory study has some limitations, such as a limited number of patients, little information on patients’ dietary pattern, or the relatively low level of taxonomic resolution of 16S rRNA gene metagenomics, we obtained innovative and interesting preliminary data, such as the same duodenal microbial architecture between aCD and pCD patients but differences in the serum FFA’s profiles and in the duodenal adaptive immunity. However, we will try to overcome the mentioned limits in a future larger and more detailed investigation with aCD and pCD patients, divided into homogeneous groups considering also age, gender, nutrition, and comorbidity.

Although the precise relationship between host lipid metabolism and gut microbiota remains to be clarified, our findings suggest a possible modification in microbial functionality that needs to be further explored assessing also the contribution of the colonic microbiota, in order to detect potential new biomarkers of human pCD.

## Data availability statement

The data presented in this study are deposited in the NCBI Gene Expression Omnibus repository, accession number: GSE181070 and the analysis script is available at https://github.com/matteoramazzotti/papers/tree/main/2021celiac.

## Ethics statement

The studies involving human participants were reviewed and approved by Ethical Committee of the University of Florence, Italy (CE: 10443_oss, 14/02/2017). The patients/participants provided their written informed consent to participate in this study. Written informed consent was obtained from the individual(s) for the publication of any potentially identifiable images or data included in this article.

## Author contributions

ASC, AA, and ER designed the study. FR, GN, and GL collected the samples. FR, DR, SB, MP, MM, and GB performed the experiments. FR, ER, SB, and EN analyzed the data. MC and MR analyzed microbiota data. ER and FR wrote the manuscript. ER edited the manuscript. AA, DR, and ASC supervised the manuscript. ER, AA, and ASC provided for funding acquisition. All authors have approved the final draft submitted.

## References

[B1] Abdukhakimova D. Dossybayeva K. Poddighe D. (2021). Fecal and duodenal microbiota in pediatric celiac disease. *Front. Pediatr.* 9:652208. 10.3389/fped.2021.652208 33968854 PMC8100229

[B2] Aghdassi E. Ma D. W. Morrison S. Hillyer L. M. Clarke S. Gladman D. D. (2011). Alterations in circulating fatty acid composition in patients with systemic lupus erythematosus: A pilot study. *JPEN J. Parenter. Enteral. Nutr.* 35 198–208. 10.1177/0148607110386378 21378249

[B3] Akobeng A. K. Singh P. Kumar M. Al Khodor S. (2020). Role of the gut microbiota in the pathogenesis of coeliac disease and potential therapeutic implications. *Eur. J. Nutr.* 59 3369–3390. 10.1007/s00394-020-02324-y 32651763 PMC7669811

[B4] Albanese D. Fontana P. De Filippo C. Cavalieri D. Donati C. (2015). MICCA: A complete and accurate software for taxonomic profiling of metagenomic data. *Sci. Rep.* 5:9743. 10.1038/srep09743 25988396 PMC4649890

[B5] Anderson R. P. (2020). Innate and adaptive immunity in celiac disease. *Curr. Opin. Gastroenterol.* 36 470–478. 10.1097/MOG.0000000000000672 32889822

[B6] Atarashi K. Tanoue T. Oshima K. Suda W. Nagano Y. Nishikawa H. (2013). Treg induction by a rationally selected mixture of clostridia strains from the human microbiota. *Nature* 500 232–236. 10.1038/nature12331 23842501

[B7] Auricchio R. Tosco A. Piccolo E. Galatola M. Izzo V. Maglio M. (2014). Potential celiac children: 9-year follow-up on a gluten-containing diet. *Am. J. Gastroenterol.* 109 913–921. 10.1038/ajg.2014.77 24777149

[B8] Baldi S. Menicatti M. Nannini G. Niccolai E. Russo E. Ricci F. (2021). Free fatty acids signature in human intestinal disorders: Significant association between butyric acid and celiac disease. *Nutrients* 13:742. 10.3390/nu13030742 33652681 PMC7996737

[B9] Bernini P. Bertini I. Calabro A. la Marca G. Lami G. Luchinat C. (2011). Are patients with potential celiac disease really potential? The answer of metabonomics. *J. Proteome Res.* 10 714–721. 10.1021/pr100896s 21090607

[B10] Bertini I. Calabro A. De Carli V. Luchinat C. Nepi S. Porfirio B. (2009). The metabonomic signature of celiac disease. *J. Proteome Res.* 8 170–177. 10.1021/pr800548z 19072164

[B11] Biagi F. Trotta L. Alfano C. Balduzzi D. Staffieri V. Bianchi P. I. (2013). Prevalence and natural history of potential celiac disease in adult patients. *Scand. J. Gastroenterol.* 48 537–542. 10.3109/00365521.2013.777470 23506211

[B12] Braniste V. Al-Asmakh M. Kowal C. Anuar F. Abbaspour A. Toth M. (2014). The gut microbiota influences blood-brain barrier permeability in mice. *Sci. Transl. Med.* 6:263ra158. 10.1126/scitranslmed.3009759 25411471 PMC4396848

[B13] Brestoff J. R. Artis D. (2013). Commensal bacteria at the interface of host metabolism and the immune system. *Nat. Immunol.* 14 676–684. 10.1038/ni.2640 23778795 PMC4013146

[B14] Buscarinu M. C. Cerasoli B. Annibali V. Policano C. Lionetto L. Capi M. (2017). Altered intestinal permeability in patients with relapsing-remitting multiple sclerosis: A pilot study. *Mult. Scler.* 23 442–446. 10.1177/1352458516652498 27270497

[B15] Cheng J. Kalliomaki M. Heilig H. G. Palva A. Lahteenoja H. de Vos W. M. (2013). Duodenal microbiota composition and mucosal homeostasis in pediatric celiac disease. *BMC Gastroenterol.* 13:113. 10.1186/1471-230X-13-113 23844808 PMC3716955

[B16] Ciccocioppo R. Di Sabatino A. Corazza G. R. (2005). The immune recognition of gluten in coeliac disease. *Clin. Exp. Immunol.* 140 408–416. 10.1111/j.1365-2249.2005.02783.x 15932501 PMC1809391

[B17] Cukrowska B. Sowinska A. Bierla J. B. Czarnowska E. Rybak A. Grzybowska-Chlebowczyk U. (2017). Intestinal epithelium, intraepithelial lymphocytes and the gut microbiota – key players in the pathogenesis of celiac disease. *World J. Gastroenterol.* 23 7505–7518. 10.3748/wjg.v23.i42.7505 29204051 PMC5698244

[B18] Dai L. Goncalves C. M. Lin Z. Huang J. Lu H. Yi L. (2015). Exploring metabolic syndrome serum free fatty acid profiles based on GC-SIM-MS combined with random forests and canonical correlation analysis. *Talanta* 135 108–114. 10.1016/j.talanta.2014.12.039 25640133

[B19] den Besten G. van Eunen K. Groen A. K. Venema K. Reijngoud D. J. Bakker B. M. (2013). The role of short-chain fatty acids in the interplay between diet, gut microbiota, and host energy metabolism. *J. Lipid Res.* 54 2325–2340. 10.1194/jlr.R036012 23821742 PMC3735932

[B20] Ferguson A. Arranz E. O’Mahony S. (1993). Clinical and pathological spectrum of coeliac disease–active, silent, latent, potential. *Gut* 34 150–151. 10.1136/gut.34.2.150 8432463 PMC1373959

[B21] Frommer K. W. Schaffler A. Rehart S. Lehr A. Muller-Ladner U. Neumann E. (2015). Free fatty acids: Potential proinflammatory mediators in rheumatic diseases. *Ann. Rheum. Dis.* 74 303–310. 10.1136/annrheumdis-2013-203755 24285492

[B22] Frossi B. De Carli M. Calabro A. (2019). Coeliac disease and mast cells. *Int. J. Mol. Sci.* 20:3400. 10.3390/ijms20143400 31373285 PMC6678566

[B23] Gibiino G. Ianiro G. Cammarota G. Gasbarrini A. (2017). The gut microbiota: Its anatomy and physiology over a lifetime. *Minerva Gastroenterol. Dietol.* 63 329–336. 10.23736/S1121-421X.17.02405-9 28381083

[B24] Honda K. L. Lamon-Fava S. Matthan N. R. Wu D. Lichtenstein A. H. (2015). EPA and DHA exposure alters the inflammatory response but not the surface expression of toll-like receptor 4 in macrophages. *Lipids* 50 121–129. 10.1007/s11745-014-3971-y 25408476 PMC4306624

[B25] Hung A. M. Booker C. Ellis C. D. Siew E. D. Graves A. J. Shintani A. (2015). Omega-3 fatty acids inhibit the up-regulation of endothelial chemokines in maintenance hemodialysis patients. *Nephrol. Dial. Transplant.* 30 266–274. 10.1093/ndt/gfu283 25204316 PMC4309191

[B26] Husby S. Koletzko S. Korponay-Szabo I. R. Mearin M. L. Phillips A. Shamir R. (2012). European society for pediatric gastroenterology, hepatology, and nutrition guidelines for the diagnosis of coeliac disease. *J. Pediatr. Gastroenterol. Nutr.* 54 136–160. 10.1097/MPG.0b013e31821a23d0 22197856

[B27] Jabri B. Sollid L. M. (2009). Tissue-mediated control of immunopathology in coeliac disease. *Nat. Rev. Immunol.* 9 858–870. 10.1038/nri2670 19935805

[B28] Kanauchi O. Andoh A. Iwanaga T. Fujiyama Y. Mitsuyama K. Toyonaga A. (1999). Germinated barley foodstuffs attenuate colonic mucosal damage and mucosal nuclear factor kappa B activity in a spontaneous colitis model. *J. Gastroenterol. Hepatol.* 14 1173–1179. 10.1046/j.1440-1746.1999.02025.x 10634153

[B29] Karell K. Louka A. S. Moodie S. J. Ascher H. Clot F. Greco L. (2003). HLA types in celiac disease patients not carrying the DQA1*05-DQB1*02 (DQ2) heterodimer: Results from the European genetics cluster on celiac disease. *Hum. Immunol.* 64 469–477. 10.1016/S0198-8859(03)00027-2 12651074

[B30] Khosravi A. Mazmanian S. K. (2013). Disruption of the gut microbiome as a risk factor for microbial infections. *Curr. Opin. Microbiol.* 16 221–227. 10.1016/j.mib.2013.03.009 23597788 PMC5695238

[B31] Kurppa K. Collin P. Viljamaa M. Haimila K. Saavalainen P. Partanen J. (2009). Diagnosing mild enteropathy celiac disease: A randomized, controlled clinical study. *Gastroenterology* 136 816–823. 10.1053/j.gastro.2008.11.040 19111551

[B32] Lionetti E. Castellaneta S. Pulvirenti A. Tonutti E. Francavilla R. Fasano A. (2012). Prevalence and natural history of potential celiac disease in at-family-risk infants prospectively investigated from birth. *J. Pediatr.* 161 908–914. 10.1016/j.jpeds.2012.05.008 22704250

[B33] Love M. I. Huber W. Anders S. (2014). Moderated estimation of fold change and dispersion for RNA-seq data with DESeq2. *Genome Biol.* 15:550. 10.1186/s13059-014-0550-8 25516281 PMC4302049

[B34] Ludvigsson J. F. Leffler D. A. Bai J. C. Biagi F. Fasano A. Green P. H. (2013). The Oslo definitions for coeliac disease and related terms. *Gut* 62 43–52. 10.1136/gutjnl-2011-301346 22345659 PMC3440559

[B35] Maiuri L. Picarelli A. Boirivant M. Coletta S. Mazzilli M. C. De Vincenzi M. (1996). Definition of the initial immunologic modifications upon in vitro gliadin challenge in the small intestine of celiac patients. *Gastroenterology* 110 1368–1378. 10.1053/gast.1996.v110.pm8613040 8613040

[B36] McMurdie P. J. Holmes S. (2013). phyloseq: An R package for reproducible interactive analysis and graphics of microbiome census data. *PLoS One* 8:e61217. 10.1371/journal.pone.0061217 23630581 PMC3632530

[B37] Mizuno M. Noto D. Kaga N. Chiba A. Miyake S. (2017). The dual role of short fatty acid chains in the pathogenesis of autoimmune disease models. *PLoS One* 12:e0173032. 10.1371/journal.pone.0173032 28235016 PMC5325617

[B38] Monteleone I. Sarra M. Del Vecchio Blanco G. Paoluzi O. A. Franze E. Fina D. (2010). Characterization of IL-17A-producing cells in celiac disease mucosa. *J. Immunol.* 184 2211–2218. 10.4049/jimmunol.0901919 20061410

[B39] Mucida D. Salek-Ardakani S. (2009). Regulation of TH17 cells in the mucosal surfaces. *J. Allergy Clin. Immunol.* 123 997–1003. 10.1016/j.jaci.2009.03.016 19362732 PMC2679861

[B40] Nankova B. B. Agarwal R. MacFabe D. F. La Gamma E. F. (2014). Enteric bacterial metabolites propionic and butyric acid modulate gene expression, including CREB-dependent catecholaminergic neurotransmission, in PC12 cells–possible relevance to autism spectrum disorders. *PLoS One* 9:e103740. 10.1371/journal.pone.0103740 25170769 PMC4149359

[B41] Niccolai E. Ricci F. Russo E. Nannini G. Emmi G. Taddei A. (2017). The different functional distribution of “Not Effector” T cells (treg/tnull) in colorectal cancer. *Front. Immunol.* 8:1900. 10.3389/fimmu.2017.01900 29375559 PMC5770731

[B42] Niccolai E. Russo E. Baldi S. Ricci F. Nannini G. Pedone M. (2020). Significant and conflicting correlation of IL-9 with *Prevotella* and *Bacteroides* in human colorectal cancer. *Front. Immunol.* 11:573158. 10.3389/fimmu.2020.573158 33488574 PMC7820867

[B43] Nishi S. K. Kendall C. W. Bazinet R. P. Bashyam B. Ireland C. A. Augustin L. S. (2014). Nut consumption, serum fatty acid profile and estimated coronary heart disease risk in type 2 diabetes. *Nutr. Metab. Cardiovasc. Dis.* 24 845–852. 10.1016/j.numecd.2014.04.001 24925120

[B44] Nistal E. Caminero A. Vivas S. Ruiz de Morales J. M. Saenz de Miera L. E. Rodriguez-Aparicio L. B. (2012). Differences in faecal bacteria populations and faecal bacteria metabolism in healthy adults and celiac disease patients. *Biochimie* 94 1724–1729. 10.1016/j.biochi.2012.03.025 22542995

[B45] Oberhuber G. Granditsch G. Vogelsang H. (1999). The histopathology of coeliac disease: Time for a standardized report scheme for pathologists. *Eur. J. Gastroenterol. Hepatol.* 11 1185–1194. 10.1097/00042737-199910000-00019 10524652

[B46] Pagliai G. Russo E. Niccolai E. Dinu M. Di Pilato V. Magrini A. (2020). Influence of a 3-month low-calorie Mediterranean diet compared to the vegetarian diet on human gut microbiota and SCFA: The CARDIVEG Study. *Eur. J. Nutr.* 59 2011–2024. 10.1007/s00394-019-02050-0 31292752

[B47] Pinkosky S. L. Scott J. W. Desjardins E. M. Smith B. K. Day E. A. Ford R. J. (2020). Long-chain fatty acyl-CoA esters regulate metabolism via allosteric control of AMPK beta1 isoforms. *Nat. Metab.* 2 873–881. 10.1038/s42255-020-0245-2 32719536 PMC7502547

[B48] R Development Core Team (2014). *R: A language and environment for statistical computing*. Vienna: R Foundation for Statistical Computing.

[B49] Rodriguez-Carrio J. Alperi-Lopez M. Lopez P. Ballina-Garcia F. J. Suarez A. (2016). Non-esterified fatty acids profiling in rheumatoid arthritis: Associations with clinical features and Th1 response. *PLoS One* 11:e0159573. 10.1371/journal.pone.0159573 27487156 PMC4972416

[B50] Rubio-Tapia A. Ludvigsson J. F. Brantner T. L. Murray J. A. Everhart J. E. (2012). The prevalence of celiac disease in the United States. *Am. J. Gastroenterol.* 107 1538–1544; quiz 1537, 1545. 10.1038/ajg.2012.219 22850429

[B51] Russo E. Giudici F. Ricci F. Scaringi S. Nannini G. Ficari F. (2021). Diving into inflammation: A pilot study exploring the dynamics of the immune-microbiota axis in ileal tissue layers of patients with Crohn’s disease. *J. Crohns Colitis* 15 1500–1516. 10.1093/ecco-jcc/jjab034 33611347

[B52] Schonfeld P. Wojtczak L. (2016). Short- and medium-chain fatty acids in energy metabolism: The cellular perspective. *J. Lipid Res.* 57 943–954. 10.1194/jlr.R067629 27080715 PMC4878196

[B53] Thursby E. Juge N. (2017). Introduction to the human gut microbiota. *Biochem. J.* 474 1823–1836. 10.1042/BCJ20160510 28512250 PMC5433529

[B54] Tosco A. Salvati V. M. Auricchio R. Maglio M. Borrelli M. Coruzzo A. (2011). Natural history of potential celiac disease in children. *Clin. Gastroenterol. Hepatol.* 9 320–325; quiz e336. 10.1016/j.cgh.2010.09.006 20851213

[B55] Troncone R. Greco L. Mayer M. Paparo F. Caputo N. Micillo M. (1996). Latent and potential coeliac disease. *Acta Paediatr. Suppl.* 412 10–14. 10.1111/j.1651-2227.1996.tb14240.x 8783748

[B56] Valdes A. M. Walter J. Segal E. Spector T. D. (2018). Role of the gut microbiota in nutrition and health. *BMJ* 361:k2179. 10.1136/bmj.k2179 29899036 PMC6000740

[B57] van Bergen J. Mulder C. J. Mearin M. L. Koning F. (2015). Local communication among mucosal immune cells in patients with celiac disease. *Gastroenterology* 148 1187–1194. 10.1053/j.gastro.2015.01.030 25623043

[B58] van Hees N. J. Giltay E. J. Geleijnse J. M. Janssen N. van der Does W. (2014). DHA serum levels were significantly higher in celiac disease patients compared to healthy controls and were unrelated to depression. *PLoS One* 9:e97778. 10.1371/journal.pone.0097778 24841484 PMC4026409

[B59] Vignoli A. Orlandini B. Tenori L. Biagini M. R. Milani S. Renzi D. (2019). Metabolic signature of primary biliary cholangitis and its comparison with celiac disease. *J. Proteome Res.* 18 1228–1236. 10.1021/acs.jproteome.8b00849 30539636

[B60] Vitale S. Santarlasci V. Camarca A. Picascia S. Pasquale A. D. Maglio M. (2019). The intestinal expansion of TCRgammadelta(+) and disappearance of IL4(+) T cells suggest their involvement in the evolution from potential to overt celiac disease. *Eur. J. Immunol.* 49 2222–2234. 10.1002/eji.201948098 31553811

[B61] Voisine J. Abadie V. (2021). Interplay between gluten, HLA, innate and adaptive immunity orchestrates the development of coeliac disease. *Front. Immunol.* 12:674313. 10.3389/fimmu.2021.674313 34149709 PMC8206552

[B62] Willis A. Bunge J. (2015). Estimating diversity via frequency ratios. *Biometrics* 71 1042–1049. 10.1111/biom.12332 26038228

[B63] Woo V. Alenghat T. (2017). Host-microbiota interactions: Epigenomic regulation. *Curr. Opin. Immunol.* 44 52–60. 10.1016/j.coi.2016.12.001 28103497 PMC5451311

[B64] Yuille S. Reichardt N. Panda S. Dunbar H. Mulder I. E. (2018). Human gut bacteria as potent class I histone deacetylase inhibitors in vitro through production of butyric acid and valeric acid. *PLoS One* 13:e0201073. 10.1371/journal.pone.0201073 30052654 PMC6063406

[B65] Zanini B. Caselani F. Magni A. Turini D. Ferraresi A. Lanzarotto F. (2013). Celiac disease with mild enteropathy is not mild disease. *Clin. Gastroenterol. Hepatol.* 11 253–258. 10.1016/j.cgh.2012.09.027 23022697

