# Corrigendum: Characterization of the “gut microbiota-immunity axis” and microbial lipid metabolites in atrophic and potential celiac disease

**DOI:** 10.3389/fmicb.2023.1165150

**Published:** 2023-05-12

**Authors:** Federica Ricci, Edda Russo, Daniela Renzi, Simone Baldi, Giulia Nannini, Gabriele Lami, Marta Menicatti, Marco Pallecchi, Gianluca Bartolucci, Elena Niccolai, Matteo Cerboneschi, Serena Smeazzetto, Matteo Ramazzotti, Amedeo Amedei, Antonino Salvatore Calabrò

**Affiliations:** ^1^Department of Biomedical, Experimental and Clinical Sciences “Mario Serio” University of Florence, Tuscany Regional Referral Center for Adult Celiac Disease, Florence, Italy; ^2^Department of Experimental and Clinical Medicine, University of Florence, Florence, Italy; ^3^Department of Neurosciences, Psychology, Drug Research and Child Health (NEUROFARBA), University of Florence, Florence, Italy; ^4^Department of Biomedical, Experimental and Clinical Sciences “Mario Serio” University of Florence, Florence, Italy

**Keywords:** potential celiac disease, celiac disease, microbiota, immune response, fatty acids, T cells, cytokines

In the published article the author names were written in the incorrect order of given and family name. The author name order has been corrected.

In the published article affiliation 3 was incorrect. Affiliation 3 has been corrected.

The Copyright has been corrected as follows:

© 2023 Ricci, Russo, Renzi, Baldi, Nannini, Lami, Menicatti, Pallecchi, Bartolucci, Niccolai, Cerboneschi, Smeazzetto, Ramazzotti, Amedei and Calabrò. This is an open-access article distributed under the terms of the Creative Commons Attribution License (CC BY). The use, distribution or reproduction in other forums is permitted, provided the original author(s) and the copyright owner(s) are credited and that the original publication in this journal is cited, in accordance with accepted academic practice. No use, distribution or reproduction is permitted which does not comply with these terms.

The “Author contributions” has been corrected as follows:

## Author contributions

ASC, AA, and ER designed the study. FR, GN, and GL collected the samples. FR, DR, SB, MP, MM, and GB performed the experiments. FR, ER, SB, and EN analyzed the data. MC and MR analyzed microbiota data. ER and FR wrote the manuscript. ER edited the manuscript. AA, DR, and ASC supervised the manuscript. ER, AA, and ASC provided for funding acquisition. All authors have approved the final draft submitted.

The authors apologize for this error and state that this does not change the scientific conclusions of the article in any way. The original article has been updated.

