# Supplementary material for: Characterization of the “gut microbiota-immunity axis” and microbial lipid metabolites in atrophic and potential celiac disease
Source: Front Microbiol. 2022 Sep 30;13:886008. doi: 10.3389/fmicb.2022.886008 (PMC9561818; doi:10.3389/fmicb.2022.886008)

SUPPLEMENTARY DATA

Figure S1. Output pf the reads preprocessing and quality control

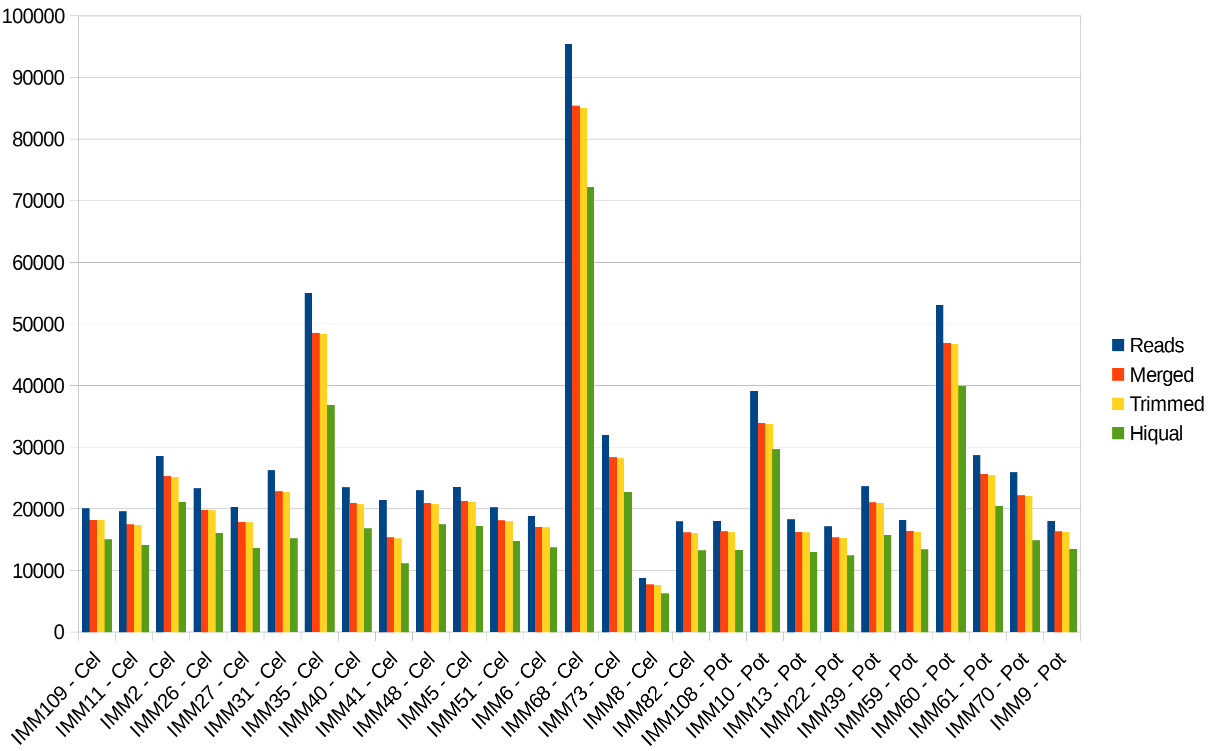

Figure S2. Saturation of samples by rarefaction analysis on OTUs

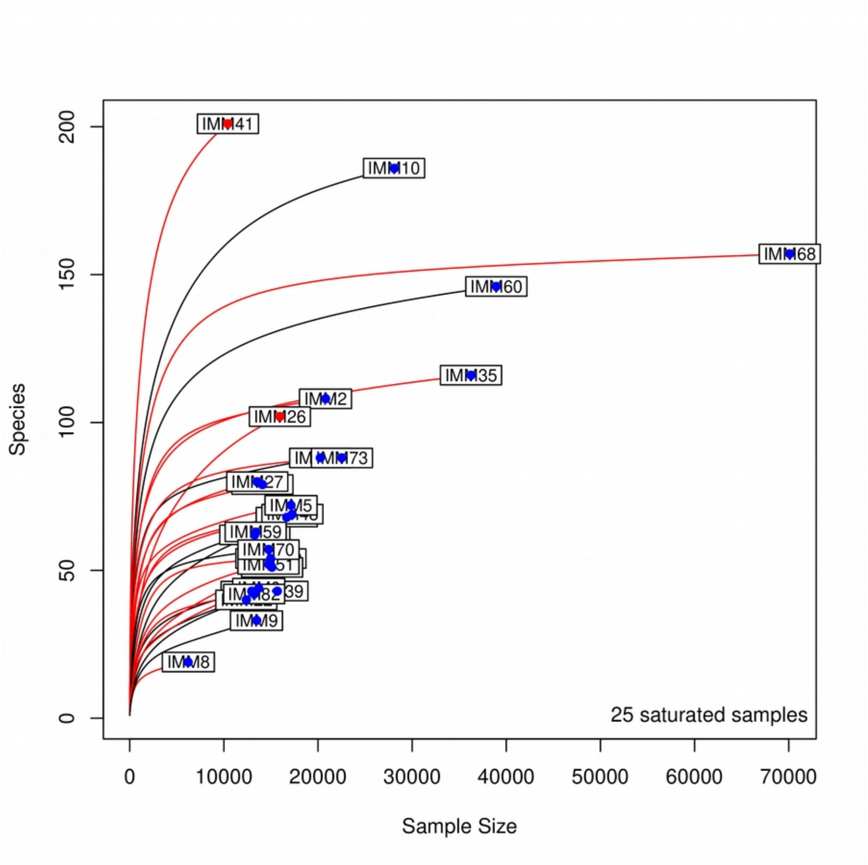

Supplement: Supplementary file 1 [file Data_Sheet_1.PDF]
